# Prevalence of *Erysipelothrix rhusiopathiae* in tonsils of domestic pigs and wild boars in Sweden

**DOI:** 10.1186/s13028-026-00881-6

**Published:** 2026-09-02

**Authors:** Mate Zoric, Robert Söderlund, Elisabeth Bagge, Helena Eriksson

**Affiliations:** 1 Department of Animal Health and Antimicrobial Strategies, Swedish Veterinary Agency, Uppsala, SE-751 89 Sweden; 2 Department of Microbiology, Swedish Veterinary Agency, Uppsala, SE-751 89 Sweden; 3 Department of Clinical Sciences, Faculty of Veterinary Medicine and Animal Sciences, Swedish University of Agricultural Sciences, Box 7054, Uppsala, SE-75007 Sweden

**Keywords:** Asymptomatic carriage, MALDI-TOF MS, Swine, Whole-genome sequencing, Zoonosis

## Abstract

*Erysipelothrix rhusiopathiae* (ER) causes erysipelas in multiple animal species and may persist in the environment or be carried asymptomatically. It is estimated that 30–50% of apparently healthy or convalescent pigs harbour ER in their tonsils and other lymphoid tissues. This study aimed to determine the prevalence of ER in the tonsils of healthy Swedish fattening pigs and wild boars. Tonsils were collected from 200 fattening pigs at slaughter from ten abattoirs across Sweden in 2017, with one pig per herd sampled. Wild boars (*n* = 180) were sampled during hunting, primarily in Östergötland County, in 2018. Cultures were performed using selective media and isolates were confirmed as ER by MALDI-TOF MS. ER was recovered from 6/200 pig tonsils (3.0%), all originating from three abattoirs in southern Sweden. ER was isolated from 76/167 (45.5%) of wild boar tonsils. Whole-genome sequencing revealed a high genetic diversity among the isolates with no dominant clones. Overall, these results indicate that Swedish pig husbandry, characterized by indoor rearing of fattening pigs, age-segregated rearing, sow vaccination, enhanced biosecurity, and restricted straw access largely prevents tonsillar colonization by ER aligning with the low occurrence of clinically diagnosed erysipelas in such herds. For wild boars, the high isolation rate suggests that wild boar could act as a reservoir and potential source of infection for domestic pigs. The potential zoonotic risk should also be considered.

**Supplementary Information:**

The online version contains supplementary material available at https://doi.org/10.1186/s13028-026-00881-6.

## Findings

Erysipelas, caused by the bacterium *Erysipelothrix rhusiopathiae* (ER), occurs in pigs worldwide and may affect various other species, including humans. Human infections are uncommon and mainly associated with occupational exposure, such as farming and slaughterhouse work [1–3].

In domestic pigs (*Sus scrofa domesticus*), erysipelas typically presents as an acute febrile disease with characteristic cutaneous lesions. A subacute form produces milder clinical signs, whereas a chronic form is mainly associated with arthritis and endocarditis [4, 5]. The bacterium is highly susceptible to penicillin, and early antibiotic treatment usually results in recovery within 24–36 h. Vaccination provides effective protection for 6–12 months when correctly administered [4]. Carriers and pigs with acute erysipelas may shed the organism in their excretions (urine, faeces) and secretions (saliva, nasal mucus) for extended periods of time, and ER can persist in the environment for prolonged periods, allowing indirect transmission [2, 4, 6].

Earlier studies have suggested that 30–50% of clinically healthy pigs may carry ER, particularly in the tonsils and other lymphoid tissues, thereby serving as a reservoir [5, 7]. However, a Swiss study reported a very low prevalence, with all 250 tonsils samples from healthy pigs at slaughter testing negative [8].

Erysipelas is not notifiable in Sweden, and comprehensive national incidence data are lacking. Nevertheless, sporadic outbreaks are reported, particularly in outdoor finishing systems, likely due to the environmental persistence of this ubiquitous bacterium [9].

The wild boar (*Sus scrofa*) is a close relative of the domestic pig. Farmed wild boar may be affected by septicaemic erysipelas [10, 11]. In addition, serological studies conducted in several European countries, including Poland, Spain and Sweden, have indicated that wild boar are exposed to ER [12–14]. However, little is known about the carrier state of ER in wild boars. An Italian study found that ER could be isolated from 34.2% of tonsils from hunted free-living wild boars [15]. We investigated tonsillar ER in healthy domestic pigs and wild boars in Sweden and compared the recovered isolates.

**Fig. 1:**
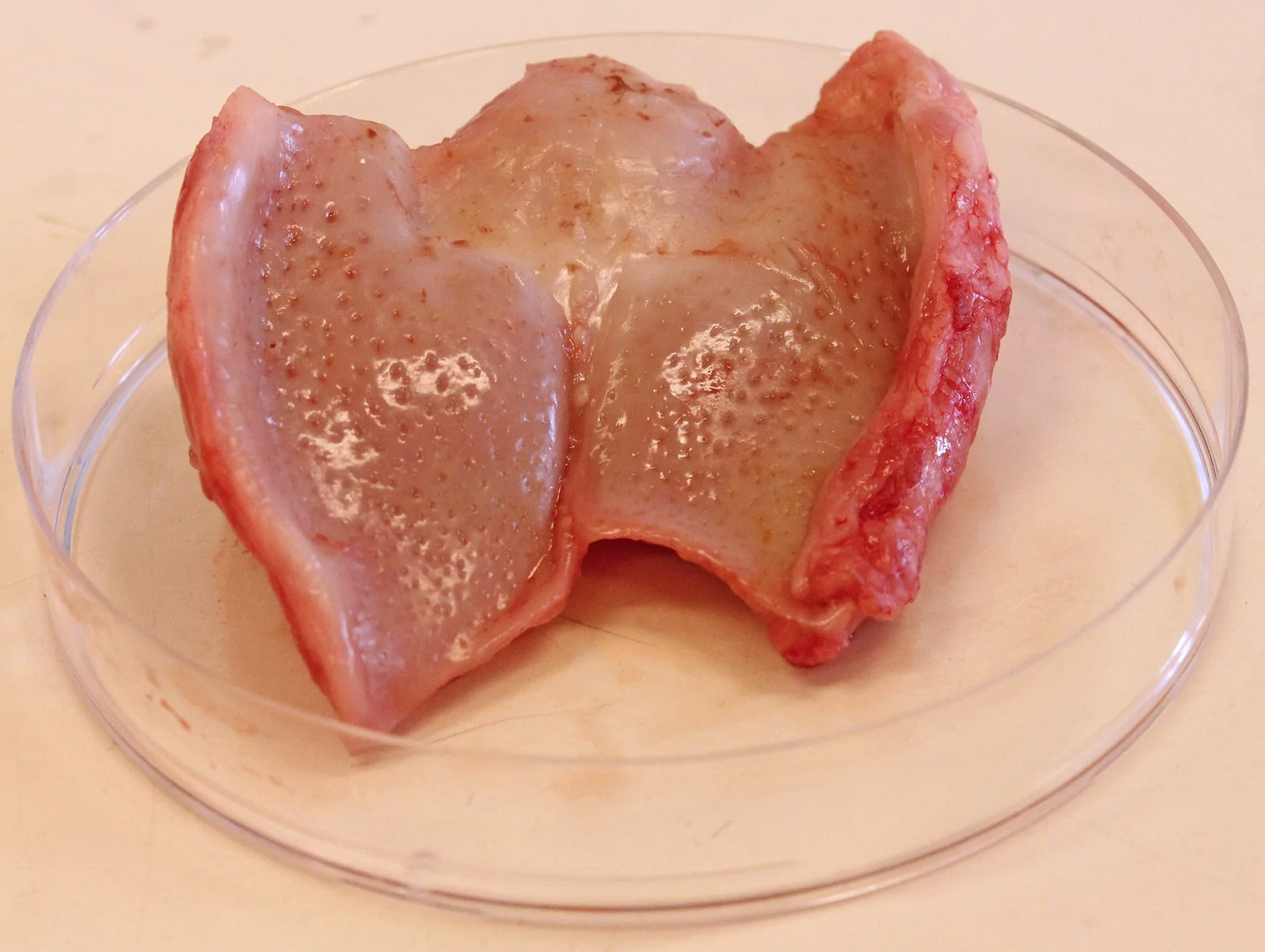
Tonsils submitted to SVA for culture for Erysipelothrix rhusiopathiae. Photo: Annika Karlsson / SVA

To assess whether healthy Swedish domestic pigs carry ER in their tonsils, tonsil samples (Fig. 1) were collected from 200 apparently healthy fattening pigs at slaughter in 2017, with 100 in the spring and 100 in the autumn. Samples were aseptically collected by official veterinarians at 10 abattoirs, which in 2016 were reported to slaughter 88% of all pigs in Sweden according to statistics from the Swedish Board of Agriculture. The number of samples per abattoir was proportional to slaughter volume in 2016, and one pig per herd was sampled.

**Fig. 2:**
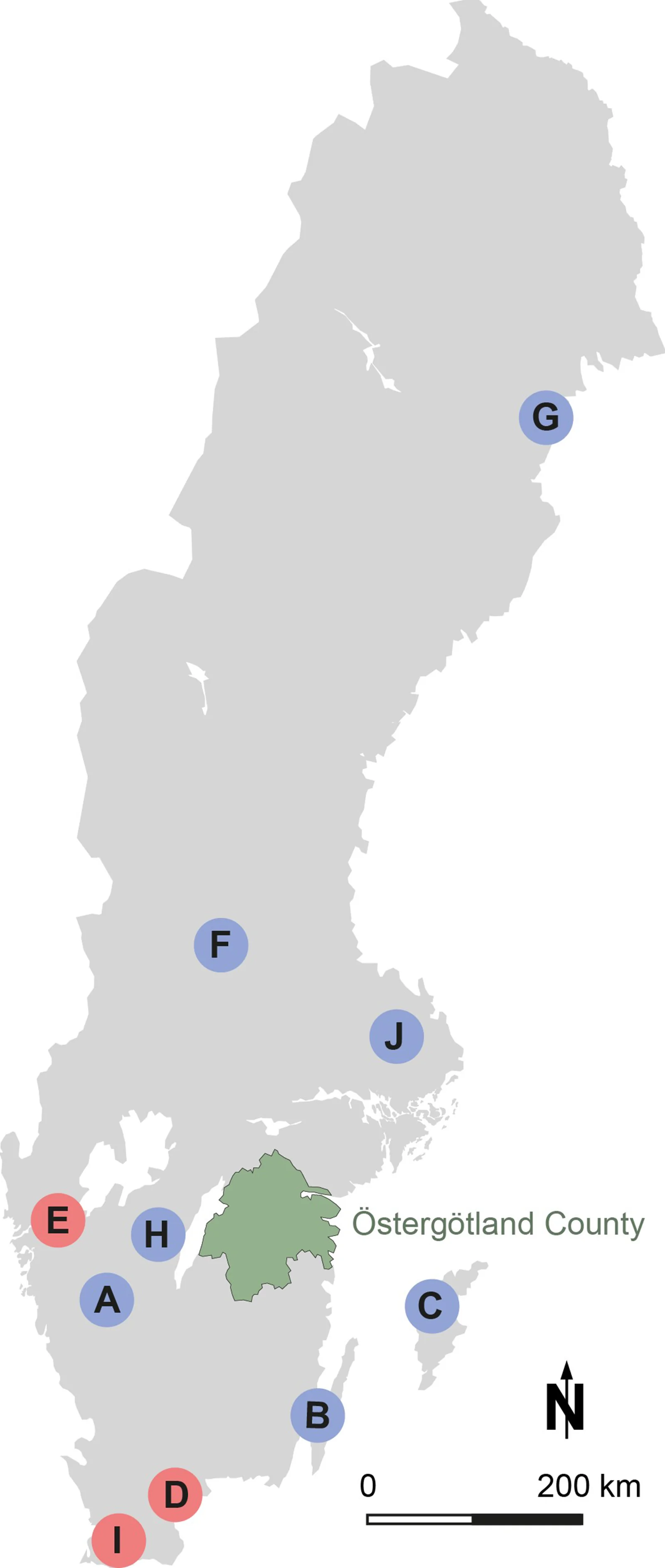
Sampling locations. Swedish abattoirs tested for *Erysipelothrix rhusiopathiae* in pig tonsils. Red circles indicate positive abattoirs and blue circles indicate negative abattoirs. Östergötland County is highlighted in green, indicating the area where sampling of hunted wild boars was concentrated

Sampling of wild boars was concentrated in Östergötland County, located in southeastern Sweden, a region with a high density of pig farms, where hunters volunteered to collect tonsil samples from wild boars hunted in 2018 (Fig. 2). For hunted wild boar in the study, data on geographical location (municipality level), sex, age group (piglet, yearling or adult) and slaughter weight were collected.

Tonsil samples were sent from abattoirs and hunters by overnight mail to the Swedish Veterinary Agency (SVA). For bacteriological analysis, an approximately 1 × 1 cm piece of tissue (Fig. 1) was inoculated in 5 mL broth containing 0.2 mg/mL sodium azide and 5 µg/mL crystal violet, and incubated at 37 °C for 48 h. One loopful (approximately 10 µL) of the broth was subsequently spread on horse blood agar plates containing 400 µg/mL kanamycin and 50 µg/mL neomycin and incubated at 37 °C for 48 h [16]. Growth of ER was confirmed by colony morphology and MALDI-TOF MS (Bruker Daltonics, Bremen, Germany).

All isolates recovered from fattening pigs (*n* = 6), together with a convenience sample of isolates from wild boar (*n* = 28), were whole-genome sequenced using the Illumina MiSeq platform. Data accession numbers and metadata regarding sequenced isolate sample origins are presented in Additional file 1. Certain sequenced isolates were derived from samples submitted with incomplete referrals and were therefore not otherwise included in the analysis described elsewhere in this report. The resulting sequence data were used for genomic comparison of isolates from multiple European countries as previously described [17]. Serotyping by in silico PCR was repeated for the present publication with primers targeting a wider range of ER serotypes [18].

Out of 200 tonsil samples from fattening pigs, ER was detected in six (3%), coming from three different abattoirs. At the abattoir level, prevalence varied between 5% and 17% (Table 1; Fig. 2).

**Table 1:** Isolation of *Erysipelothrix rhusiopathiae* from pig tonsils at 10 Swedish abattoirs in 2017

Abattoir	Tonsils (*n*)	ER-isolates (*n*)	Positive (%)
A	26	0	0
B	36	0	0
C	6	0	0
D	60	3	5
E	12	2	17
F	6	0	0
G	4	0	0
H	26	0	0
I	20	1	5
J	4	0	0

Tonsils were collected at 10 abattoirs (A-J). Location of abattoirs is shown in Fig. 2

The corresponding analyses were then performed on the wild boar samples. Samples were submitted from 180 wild boars. Ten of these were from wild boar hunted in municipalities outside Östergötland. Tonsil tissues were included in 167 of these samples and 76 (45.5%) were positive for ER. As age data did not correspond with data on slaughter weight this was excluded from the study. No significant difference in ER positivity was observed between male and female wild boars (51/103, 49.5% vs. 25/64, 39.1%, respectively; χ² = 1.74, *P* = 0.19). Results at the municipality level are presented in Table 2. To the best of our knowledge, this is the first study to demonstrate the presence of ER in wild boar tonsils in northern Europe. A previous Swedish study indicated that 18% of hunted wild boar were seropositive for ER, these animals were however hunted in other parts of the country [13]. The high isolation rate suggests that wild boar may act as a reservoir of ER thereby constituting a potential risk for domestic animals, especially those reared with outdoor access or under less stringent biosecurity.

**Table 2:** Isolation of *Erysipelothrix rhusiopathiae* from tonsils from hunted Swedish wild boar in 2018

Municipality/County	Slaughter weight ≤ 30 kg				Slaughter weight ≤ 30 kg				Total tonsils (n)
	Male		Female		Male		Female		
	Tonsils (n)	ER-isolates (n)	Tonsils (n)	ER-isolates (n)	Tonsils (n)	ER-isolates (n)	Tonsils (n)	ER-isolates (n)	
Boxholm /Östergötland	1	0	0	0	0	0	0	0	1
Finspång / Östergötland	2	0	0	0	0	0	1	0	3
Katrineholm / Södermanland	2	2	0	0	0	0	0	0	2
Linköping /Östergötland	8	3	3	0	14	7	12	4	37
Norrköping /Östergötland	17	10	7	4	24	14	14	8	62
Nyköping /Södermanland	1	0	2	1	1	0	0	0	4
Söderköping /Östergötland	12	5	9	2	17	7	12	4	50
Valdemarsvik /Östergötland	3	3	1	1	0	0	1	1	5
Västervik /Kalmar	0	0	0	0	1	0	2	0	3
Total (n)	46	23	22	8	57	28	42	17	
Total tonsils (n)									167

To further investigate the relationship between isolates from the two host populations, their genomic characteristics were compared. Overall, both pig and wild boar isolates were highly diverse in terms of SNP profile, with no indication of dominant clones [17]. Wild boar isolates were predominantly clade 2 (86%) with a smaller number of clade 3 (7%) and clade 1 (7%) isolates. The most common in silico serotype among wild boars was 2 (39%) followed by 9 (21%), 5 (18%), 6, 1b, and non-typeable (each 7%). In contrast, most pig isolates were clade 1 (67%), with the remainder clade 2. Among pig isolates, 11 was the most common serotype (50%), followed by 5, 9, and 4 (each 17%). To our knowledge, ER clade 1 has not previously been reported from pigs or wild boars. Clade 1 isolates differ from other clades in virulence gene content and carry distinct variants of the Spa gene, which is involved in pathogenesis [19]. Thus, clade 1 may be widespread among wild and domestic animals but overlooked due to limited sampling of asymptomatic hosts. Serotypes 2 and 1b recovered from wild boars are considered associated with ER disease outbreaks but did not occur in domestic pigs. There was no overlap between these serotypes and clade 1. Overall, these findings demonstrate marked differences in ER prevalence and population structure between domestic pigs and wild boars.

It has previously been reported that 30–50% of healthy pigs are subclinical carriers of ER, a prevalence comparable to that observed in Swedish wild boars in the present study. In contrast, only 3% of slaughter pigs tested positive, indicating a markedly lower prevalence in domestic production. Similar findings have been reported in previous studies, where intensive indoor pig production combined with high biosecurity and vaccination was associated with reduced colonization and a lower occurrence of clinical erysipelas [20, 21]. Our findings suggest that Swedish production practices, including indoor housing, age-segregated rearing, sow vaccination, strict biosecurity measures, and limited straw usage, are associated with reduced colonization by ER. This is reflected in the low frequency of clinical erysipelas observed in Swedish pig herds.

The comparison with wild boars highlights the influence of ecological and management factors on ER epidemiology. Wild boars, exposed to diverse environmental sources and frequent contact with conspecifics, appear to maintain a higher baseline prevalence, suggesting their role as a natural reservoir for ER. They also carry a higher proportion of strains with characteristics associated with clinical disease in porcine hosts, i.e., clades 2/3 and serotypes 2/1b. In contrast, the controlled conditions in domestic pig production substantially limit opportunities for transmission, thereby minimizing both subclinical carriage and clinical disease. These findings underscore the importance of species-specific management strategies in shaping the epidemiology of ER and emphasize the effectiveness of current Swedish husbandry practices in maintaining herd health.

Although this study was not designed to investigate transmission between domestic pigs and wild boars, the occurrence of ER in both host populations highlights the wildlife–livestock interface. Transmission is likely to be most relevant in outdoor pig production. However, our data do not permit conclusions regarding transmission direction or frequency.

These findings are relevant from a public health perspective. While the low prevalence in domestic pigs suggests limited occupational risk in the Swedish pig industry, the high isolation rate in wild boars indicates a potential source of infection for hunters and others handling carcasses.

## Supplementary Information

Below is the link to the electronic supplementary material.


Supplementary Material 1


## Data Availability

No datasets were generated or analysed during the current study.

## Declarations

### Prior publication

Parts of these data were previously published in the Proceedings of the 10th European Symposium of Porcine Health Management, Barcelona, Spain, May 9–11 2018 and the Proceedings of the 11th European Symposium of Porcine Health Management, Utrecht, Netherlands, May 22–24 2019.
